# Hyperpyrexia in a previously healthy pregnant female with COVID pneumonia: a case report and review of the literature

**DOI:** 10.1186/s13256-023-04008-7

**Published:** 2023-06-24

**Authors:** Nipun Lakshitha de Silva, Amitha Fernando, Rohini Wadanambi, A. R. J. P Niyas, Nihal Munasinghe, Gnani Somasundaram

**Affiliations:** 1 Department of Clinical Sciences, Faculty of Medicine, General Sir John Kotelawala Defence University, Kandawala Estate, Ratmalana, Sri Lanka; 2 National Hospital of Sri Lanka, Colombo, Sri Lanka; 3 Lanka Hospitals, Colombo, Sri Lanka; 4 Castle Street Hospital for Women, Colombo, Sri Lanka

**Keywords:** COVID, Hyperpyrexia, SARS CoV-2

## Abstract

**Background:**

Infection due to the SARS-CoV-2 virus can have a wide range of presentations from asymptomatic/mildly symptomatic to severe disease with multiorgan failure. Fever is a common symptom. But hyperpyrexia defined as temperature > 41.5 °C is not usual in COVID-19.

**Case presentation:**

A 24-year-old previously well Sri Lankan female in the 24th week of gestation of her first pregnancy presented with fever and shortness of breath. She was confirmed to have coronavirus disease-2019 (COVID-19). History was suggestive of late presentation on approximately the eighth day of the illness. She had rapidly deteriorating hypoxia due to COVID pneumonia requiring mechanical ventilation two days after the admission. There was evidence of cytokine storm without any secondary bacterial infection. She received glucocorticoids, tocilizumab, and intravenous antibiotics. Although she initially showed mild improvements, she subsequently developed high-grade fever with the axillary temperature rising to 41.7 °C starting from the seventh day of admission. There were no causative medicines or risk factors to explain hyperpyrexia. She died on the ninth day of admission.

**Conclusions:**

There are no reports of patients developing this complication during pregnancy. The pathophysiology of this rare life-threatening complication remains elusive. Detailed reporting and in-depth analysis of such patients will facilitate the understanding of the associations and successful management of these patients.

## Background

The novel Coronavirus disease-2019 (COVID-19) global pandemic due to the severe acute respiratory syndrome coronavirus-2 (SARS-CoV-2) virus has created a massive public health crisis with its widespread transmission, morbidity, and mortality. The commonest complication is pneumonia with hypoxic respiratory failure [1]. Patients with severe disease can experience multiorgan involvement due to the cytokine storm [2]. Though fever is common in these patients, very high fever has been described sparsely.

Hyperpyrexia is defined as fever > 41.5 °C (106.7 °F) [3]. This extreme elevation is rarely observed due to infections. Malignant hyperthermia, neuroleptic malignant syndrome and hypothalamic injury are likely to cause this degree of body temperature elevation. Hyperpyrexia due to several potential causes has been described in a few, mostly middle-aged patients with COVID-19. We report a young pregnant female with COVID-19 who developed hyperpyrexia without additional causative factors.

## Case presentation

A 24-year-old Sri Lankan female in her 24th week of gestation presented with a one-day history of fever and shortness of breath. She had preceding generalised body aches and lethargy for one week. These symptoms were overlooked by the patient and family members. On the day of the presentation, she experienced rapidly progressing dyspnoea. She was dyspnoeic even at rest. She had a mildly productive cough with a small amount of whitish sputum. There was no haemoptysis, chest pain or other symptoms. She was not on any medications other than vitamins that are routinely given during pregnancy.

There was no history of diabetes, hypertension, asthma, or any other comorbidity. There was no family history of malignant hyperthermia. She had not undergone any surgeries.

At the time of the presentation, she was alert and oriented. She appeared ill and dyspnoeic. Examination revealed an axillary temperature of 100 °F, respiratory rate of 28 breaths per min, arterial oxygen saturation of 90% on air, pulse rate of 100/min and blood pressure of 100/60 mmHg. Lung fields were normal on examination. Abdominal examination was compatible with a 24-week gestation. There were no other signs on examination.

Nasopharyngeal swabs were performed, revealing positive results on both the rapid antigen test and polymerase chain reaction (PCR) for SARS-CoV-2 (alpha variant). Results of the investigations at the time of admission are summarised in Table 1. Chest radiograph showed bilateral peripheral predominant consolidation. There was no evidence of bacterial infection. Evidence was in favour of late presentation with COVID-19 pneumonia. Interleukin-6 level was 362.3 pg/mL (< 4.4) suggesting a cytokine storm at the time of admission.

**Table 1:** Summary of investigations and their progress during the hospital stay

Investigation	Reference range	At the time of admission (day 1)	Day 4	At the time of hyperpyrexia (D7)	Before death (day 8)
White cell count (× 10^9^/L)	4–10	6.31	15.59	14.17	25.84
Neutrophils (× 10^9^/L)	2–7	5.62	13.88	9.92	19.64
Lymphocytes (× 10^9^/L)	1–3	0.59	1.09	3.12	3.1
Haematocrit (%)	36–46	31.6	42	31.3	38.4
Platelet count (× 10^9^/L)	150–410	169	313	232	137
C-reactive protein (mg/L)	< 3	147	46.1	4	2.1
Procalcitonin (ng/mL)	< 0.05	< 0.05	0.15	0.26	84.78
Lactate dehydrogenase (U/L)	135–250	342		970	2881
Ferritin (ng/mL)	15–150	137		438	9153
Arterial blood pH	7.35–7.45	7.32	7.41	7.36	7.21
Arterial bicarbonate (mmol/L)	22–26	20.2	26.4	29	18.3
PaO_2_/FiO_2_ (mmHg)		49.4	243	80	215
Arterial PCO_2_ (mmHg)	35–45	38.2	41	52	45.3
AST (U/L)	< 32	25	15	70	572
ALT (U/L)	< 45	9	46	33	207
Total Bilirubin (mg/dL)	0.1–1.2	0.2	0.2	0.9	2
Albumin (g/L)	35–52	34.3	35.3	30.8	28.5
Creatinine (mg/dL)	0.51–0.95	0.79	0.8	0.72	2.32
Sodium (mmol/L)	136–145	137	143	149	150
Potassium (mmol/L)	3.5–5.1	3.8	4.4	4.6	5.1
CPK (U/L)	20–180				1410
Blood culture		No growth		No growth	
Urine culture		No growth		No growth	

*ALT* alanine aminotransferase, *AST* aspartate aminotransferase, *CPK* creatine phosphokinase, *FiO*_*2*_ fractional inspired oxygen, *PaO*_*2*_ partial pressure of oxygen

She was started on low-flow oxygen via a face mask. Despite gradual increments in oxygen support, there was rapid deterioration resulting in hypoxia within the first few hours. Arterial oxygen saturation dropped to 85% on 15 L/min oxygen via face mask. She was moved to the intensive care unit and supported with continuous positive pressure ventilation (CPAP). She was treated with intravenous ceftriaxone 1000 mg twice daily, intravenous dexamethasone 10 mg twice daily, subcutaneous enoxaparin 60 mg twice daily and two doses of intravenous tocilizumab on the first and second days from admission.

There was a poor response to CPAP, and she was intubated and ventilated two days after admission. There was mild pyrexia during the first six days of admission. The patient showed evidence of improvement with improved chest radiograph findings and arterial oxygenation (Table 1). She remained conscious during this period and received sedation with morphine, midazolam, and paralysis with atracurium. She did not receive any antipsychotic medicines.

**Fig. 1:**
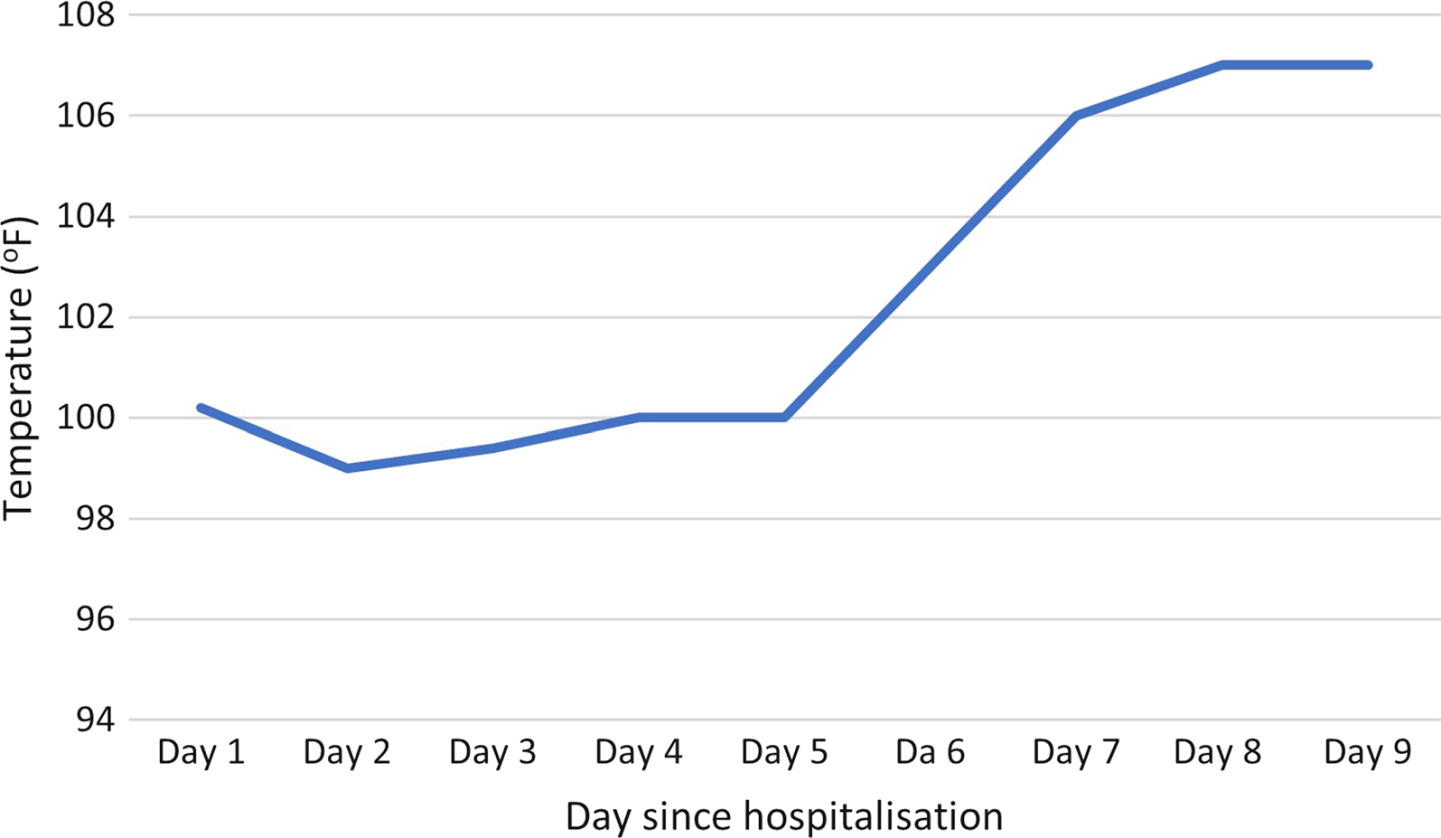
Daily axillary temperature change during the hospital stay. Figure shows that the axillary temperature of the patient was only mildly elevated during the first five days of admission. It rises from the sixth day of admission and exceeds 106.7 °F from the seventh day

On the seventh day of admission, the axillary temperature increased to 106 °F (Fig. 1). There was no evidence of bacterial infection on the first day of hyperpyrexia. She had severe tachycardia of 150 to 160 beats per min and had labile blood pressure. Examination for rigidity and reflexes was limited due to continued muscle relaxation. However, there was no increased requirement for muscle relaxants to indicate rigidity. The antibiotic regime was changed to piperacillin-tazobactam empirically and fluconazole was added. Recommended body cooling methods failed to reduce body temperature and it rose to 107 °F the next day. She was then treated with intravenous bromocriptine. She gradually deteriorated with the development of hypotension, worsening hypoxia, and acute kidney and liver injury. She died on the ninth day of admission.

## Discussion and conclusions

Fever is one of the most frequent manifestations of COVID-19 [1]. It has been recognised to be predictive of mortality in patients ventilated in intensive care with COVID-19 [4]. But hyperpyrexia, defined as fever > 41.5 °C has not been commonly observed in COVID-19 in clinical practice, nor has it been reported frequently in the existing literature. Recognised causes of hyperpyrexia include malignant hyperthermia, neuroleptic malignant syndrome, heat stroke and hypothalamic injury. Therefore, we performed a systematic literature search to identify reported patients with hyperpyrexia due to COVID-19.

A comprehensive search was done on 21/06/2020 using the search engines, PubMed and Google Scholar. The PubMed search strategy was as follows: ((covid[Title/Abstract] OR SARS[Title/Abstract])) AND ((hyperpyrexia[Title/Abstract]) OR (hyperthermia[Title/Abstract])). Similarly, a search in Google Scholar for ‘covid AND hyperpyrexia’ was performed. No language restrictions were applied. We intended to select case reports or case series describing patients with laboratory-confirmed COVID-19 and temperature > 41.5 °C.

We first screened the title and abstract to select full-text articles. After screening, eligible articles were exported into the ‘Endnote’ reference management tool and duplicates were removed. The full-text articles were screened to identify eligible papers.

**Fig. 2:**
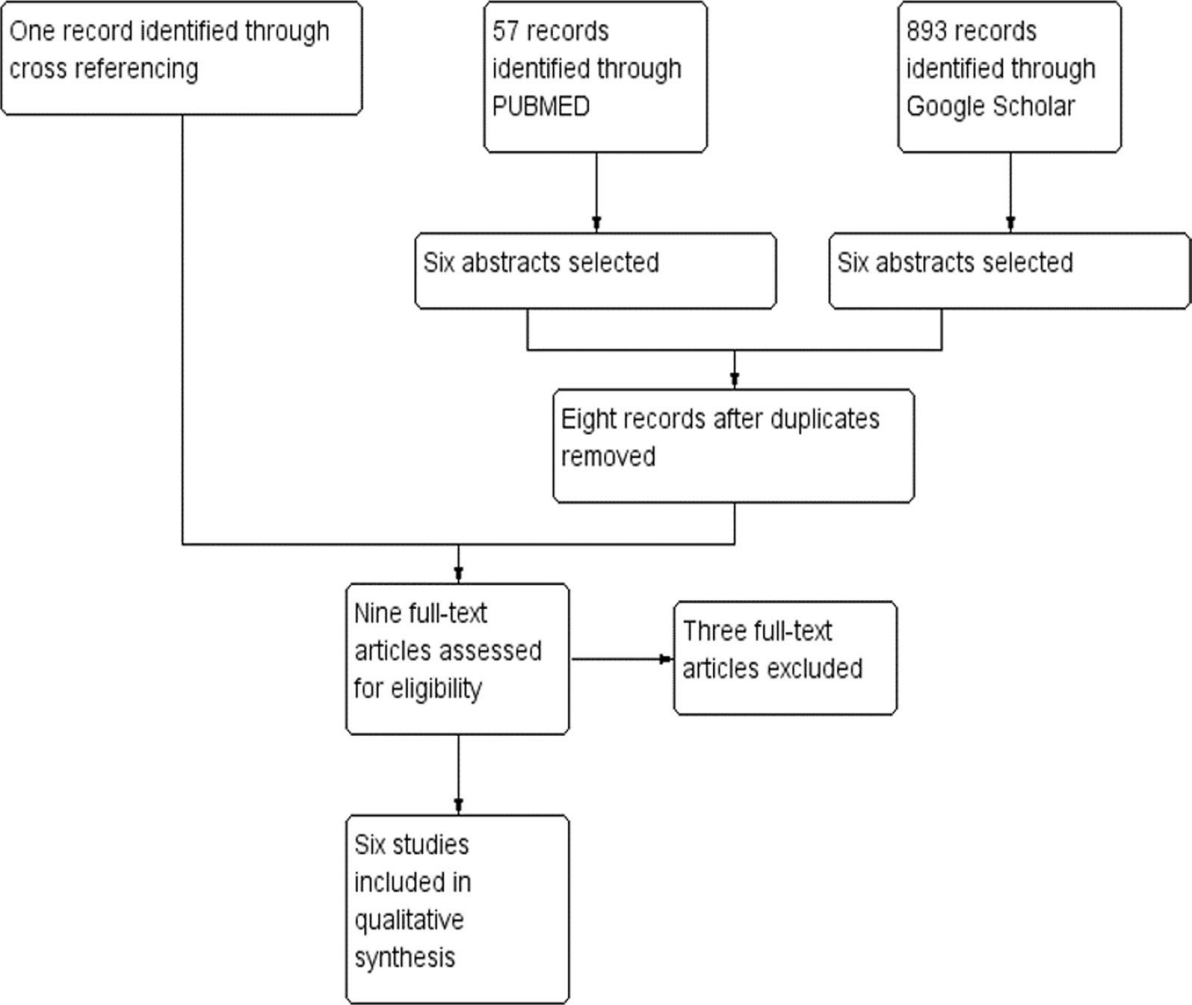
Prisma flow diagram of the literature review. The diagram shows the number of articles retrieved from data base search and cross-referencing. Out of the nine full text articles reviewed for eligibility, six studies were included in the qualitative analysis

The search in PubMed returned 57 studies. Through the screening of abstracts, six reports were selected for full-text review. The search in Google Scholar gave 893 results. After screening abstracts, six reports were selected to review the full-text article (Fig. 2). After removing duplicates, eight full-text articles were reviewed. Two articles were excluded. One describes a patient who initially recovered from SARS-Cov-2 infection and subsequently presented with fever > 40 °C. There was no clear evidence of hyperpyrexia. There were other potential causes such as invasive pulmonary aspergillosis and evidence of bacterial infection with blood culture positivity [5]. The other study is a retrospective study on hyperthermia and mortality [4]. No patients with fever exceeding > 41.5 °C were described.

An additional case report was identified after cross-referencing [6]. Though the report describes two patients with high fever, the highest reported temperatures were 41.2 °C and 40.8 °C in the two patients. Both patients developed rhabdomyolysis. Authors attribute the manifestations to drug-induced neuroleptic malignant syndrome. Potential causative agents were, midazolam, dexmedetomidine and favipiravir. Risperidone was used in one patient. Since the temperature values do not meet our criteria, this report was excluded from further analysis.

Six full-text articles describing 13 patients were eligible for detailed review (Table 2) [7–12]. All patients were confirmed with PCR for SARS-CoV-2. All the patients were receiving mechanical ventilation due to COVID pneumonia. Only four patients recovered.

**Table 2:** Summary of the six articles describing patients with hyperpyrexia and COVID-19

Author, year	Patient details	Highest temperature reported	Cause of hyperpyrexia as indicated by the authors	Additional complications	Clinical outcome	Notes
Czepiel, 2020 [7]	60-year-old female, 43-year-old female, 46-year-old male (all three had additional co-morbidities)	42.4 °C (108.3 °F) 42 °C (107.6 °F) 42 °C (107.6 °F)	High dose dexmedetomidine	Acute hypoxic respiratory failure needing intubation and ventilation (Rhabdomyolysis in the second patient)	Hyperpyrexia resolved after discontinuing dexmedetomidine (first patient died subsequently due to multiorgan failure)	Temporally associated with dexmedetomidine
Jeon, 2020 [8]	58-year-old female	41.6 °C	Hyperinflammatory syndrome of COVID (No mention about additional risk factors, was treated with lopinavir, ritonavir, glucocorticoids)	Acute hypoxic respiratory failure needing intubation and ventilation, heart failure, shock (CPK not mentioned)	Recovered	Treated with therapeutic temperature modulation and extracorporeal membrane oxygenation
Singhal, 2020 [9]	42-year-old male with diabetes mellitus	107 °F (41.7 °C)	Cytokine storm (Treated with fentanyl, midazolam, and atracurium. No mention about additional risk factors)	Acute hypoxic respiratory failure needing intubation and ventilation, elevated CPK, later multiorgan failure	Died	Pre-print
Suwanwongse, 2020 [10]	Six patients (four females), 54–62 years, five had multiple medical co-morbidities	107.2 °F (41.8 °C) − 109.7 °F (43.2 °C)	Brain injury, exuberant immune response, and thrombus formation related to COVID	All had hypoxic respiratory failure requiring intubation and ventilation (No muscle rigidity or rhabdomyolysis)	All six patients died	No exposure to presumably causative medicines
Nuckchady, 2021 [11]	20 years, female with history of asthma	44 °C	Not discussed	COVID pneumonia with hypoxia needing intubation and ventilation, initial respiratory acidosis, and subsequent metabolic alkalosis. (CPK not mentioned)	Died	Letter to the editor
Chiba, 2021 [12]	36 years, previously healthy male	41.5 °C	Malignant hyperthermia due to COVID-19, (Treated with favipiravir, midazolam, fentanyl and rocuronium. No family history of malignant hyperpyrexia, dexmedetomidine started after high fever spike)	Pneumonia with refractory hypoxia requiring mechanical ventilation, rhabdomyolysis, muscle rigidity, Systemic inflammatory response syndrome	Recovered	Improvement observed after starting treatment with dantrolene

One report describes three patients who developed hyperpyrexia temporally associated with dexmedetomidine [7]. The authors do not describe additional causative agents or exposures. However, all three patients have received hydromorphone and propofol.

The next case report describes a patient whose hyperpyrexia was attributed to the hyperinflammatory state of COVID-19 [8]. Additional risk factors were not described. Similarly, there is another case report of a patient whose hyperpyrexia was attributed to the same aetiology with no additional risk factors [9].

A case series from the United States of America describes six middle-aged patients who developed hyperpyrexia during SARS CoV-2 infection [10]. None had additional risk factors though the authors do not describe the anaesthetic agents. Authors postulate several COVID-related mechanisms to explain this phenomenon.

The only report of a patient younger than 30 years comes from Mauritius [11]. However, there is no detailed account of additional risk factors including exposure to anaesthetic agents or development of rhabdomyolysis. Another detailed account of a patient who recovered after hyperpyrexia and rhabdomyolysis was given a diagnosis of malignant hyperthermia due to COVID-19 [12].

Compared to our patient, all the others except one were older and most had comorbidities. Our patient is the first to develop hyperpyrexia due to COVID-19 during the pregnancy. Our patient also had evidence of cytokine storm and developed hypoxic respiratory failure requiring mechanical ventilation like all the patients reported before. The presence of neurological manifestations was variable in these patients. This is further compounded by the use of anaesthetic agents to support mechanical ventilation. Some developed rhabdomyolysis. Our patient also developed elevated creatine phosphokinase, though we do not have evidence to determine whether she developed rhabdomyolysis.

The exact mechanism behind this life-threatening manifestation remains unclear. As discussed above, some have been attributed to medicines like anaesthetic agents. Malignant hyperthermia, one of the commonest causes of hyperpyrexia is traditionally associated with halothane, sevoflurane, desflurane, isoflurane and the depolarizing muscle relaxant succinylcholine [13]. But none of the patients described including our patient received these agents. A family history of malignant hyperthermia was not reported in any. Another cause of hyperpyrexia is neuroleptic malignant syndrome [14]. Our patient did not receive antipsychotics before or after the admission, therefore, neuroleptic malignant syndrome becomes unlikely. Additionally, she remained conscious at the beginning of her deterioration and there was no evidence of rigidity. These contrast with the characteristic picture of neuroleptic malignant syndrome.

However, there are some concerns about dexmedetomidine causing hyperpyrexia in patients with COVID-19 [15]. Only one report describing three patients with COVID-19 shows a clear temporal association of hyperpyrexia with dexmedetomidine. Therefore, we believe that hyperpyrexia is likely to be seen in patients with severe COVID-19 with hypoxic respiratory failure. As discussed by Suwanwongse, direct brain injury due to SARS CoV-2 virus, cytokine dysregulation and thrombosis are potential mechanisms [10]. Direct brain injury to the hypothalamic thermoregulatory centre and cytokine storm are particularly likely in most of these patients. Since all the described patients were hypoxic, hypoxia-induced hypothalamic injury could also be causative.

Due to the lack of reported cases, detailed pathophysiological understanding is not possible. However, this in-depth literature review would enable clinicians to be more vigilant to recognise this rare complication in their patients. Clinicians should be encouraged to report their management strategies and patient outcome to develop effective patient management strategies for this condition with very high mortality.

## Data Availability

Not applicable.

## Abbreviations

CPAP: Continuous positive pressure ventilation
COVID-19: Coronavirus disease-2019
PCR: Polymerase chain reaction
SARS-CoV-2: Severe acute respiratory syndrome coronavirus-2
